# Bi-pedicled Visor Flap for Coverage of Two Skull Defects: A Practical Flap Option for Metastatic Scalp Lesions With a Favorable Cosmetic Outcome

**DOI:** 10.7759/cureus.40319

**Published:** 2023-06-12

**Authors:** Mohd Farid Mohd Amin, Muath Mamdouh Mahmod Al-Chalabi, Siti Fatimah Noor Mat Johar, Wan Azman Wan Sulaiman

**Affiliations:** 1 Reconstructive Sciences Unit, Universiti Sains Malaysia (USM), Kota Bharu, MYS

**Keywords:** local scalp flap, scalp flap, skull invasion, scalp reconstruction, visor flap

## Abstract

Reconstruction of the scalp after acquired defects poses a great challenge to reconstructive surgeons. In oncologic resections, the defect must be covered with well-vascularized tissue to withstand radiotherapy post-surgery. However, due to the limited scalp tissue mobility, primary closure or loco-regional flaps are challenging and limited in choice. Fortunately, with the current understanding of the robust blood supply system to the scalp tissue, they can survive with the closure under tension. In this paper, we present a case of scalp reconstruction using a bi-pedicled visor flap to cover the two skull defects after ablative surgery. In addition, this article highlights the reason for the option, the surgical procedure, and the cosmetic outcome of the surgery.

## Introduction

Scalp reconstruction can comprise a complex and diverse set of defects [1]. They can range from minor partial-thickness wounds to significant full-thickness defects involving bone and possibly dura [1]. Reconstructing any scalp defect is a challenge to the reconstructive surgeon due to several factors, such as the limited movement of the tissue, the underlying skull’s convexity, and the need to respect the hairline [2]. The closure of scalp defects is influenced by several elements, including the size and location of the defect, the quality of the remaining scalp tissue, and the hair-bearing significance [3].

Most defects after oncologic resection need well-vascularized tissue that can resist radiotherapy [4]. Therefore, the best option is either a loco-regional or a free flap [3]. However, opting for a free flap is questionable in this particular case of a primary cervical tumor with distant metastasis. These individuals are frequently not good candidates for prolonged procedures and the subsequent recovery associated with free tissue transplantation [3]. Moreover, in metastatic scalp lesions, the risk of recurrence is significant; hence, secondary surgery is later preferable for free tissue transfer [4]. Nevertheless, we have not found any literature regarding the coverage of two skull defects by the visor flap. Considering the best functional and cosmetic outcome, we performed the bi-pedicled visor flap in this case.

## Case presentation

**Figure 1:**
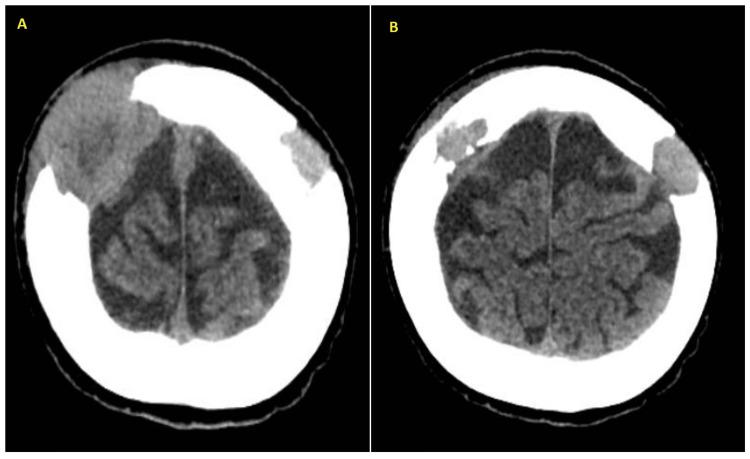
A brain CT scan shows a lytic lesion at the right parietal bone involving the inner and outer tables associated with a soft tissue component with intracranial extension (A) and a lytic lesion at the left parietal bone involving the inner and outer tables associated with a soft tissue component without intracranial extension (B).

A 49-year-old lady was diagnosed with cervical carcinoma stage IB2 and underwent a Wertheim hysterectomy and bilateral salpingo-oophorectomy to remove the primary tumor. After completing her brachytherapy, she underwent 12 cycles of chemotherapy. Unfortunately, 10 months after the surgery, she noticed two scalp swellings over the parietal region. A brain CT scan showed two lesions over the right and left parietal bones (Figure 1). The lytic lesion at the right parietal bone, involving the inner and outer tables, measured 4.4 cm × 4.5 cm and was associated with a soft tissue component measuring 4.0 cm × 5.0 cm × 4.5 cm. The lesion extended inferiorly into the intracranial region with poor demarcation with the underlying dural layer. Another lesion at the left parietal bone involving the inner and outer tables was associated with a soft tissue component measuring 2.1 cm × 1.5 cm × 1.2 cm. No intracranial extension of this lesion was noted.

**Figure 2:**
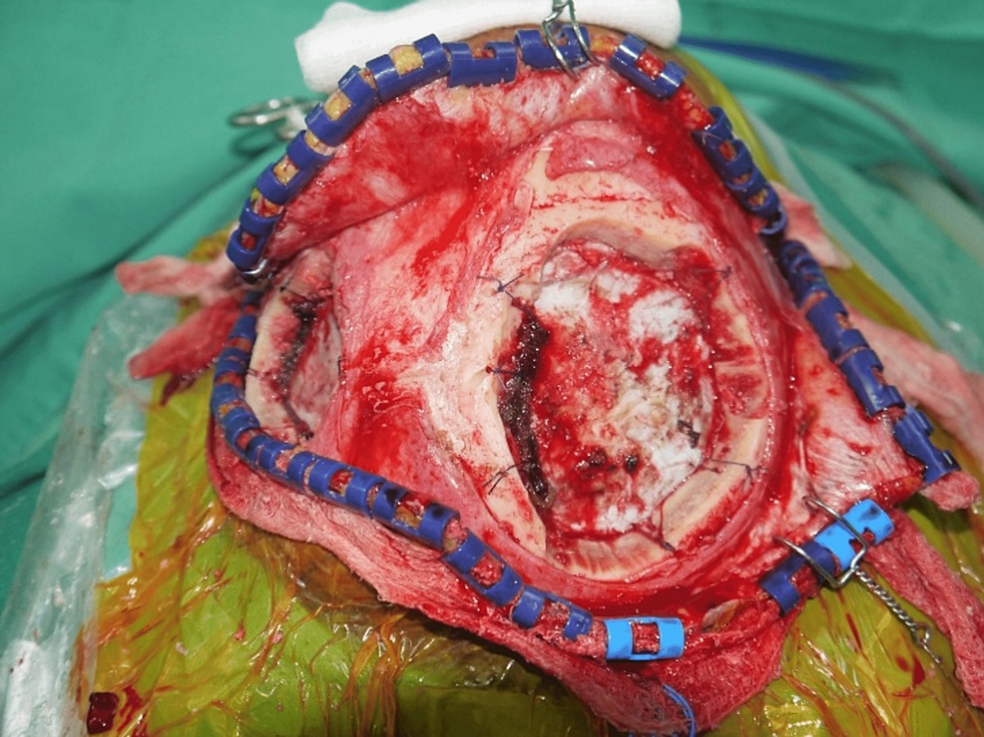
A bilateral parietal craniectomy resulting in two skull bone defects.

Subsequently, the patient was planned for tumor excision and soft tissue coverage with a loco-regional flap. Intraoperatively, the two lytic lesions were marked. A bi-pedicle visor flap was planned based on a parietal branch of the superficial temporal artery and the posterior auricular artery, identified by a handheld Doppler and marked. The neurosurgical team performed a bilateral parietal craniectomy, resulting in two skull bone defects in the parietal region, measuring 12 × 9 cm on the right parietal and 7 × 5 cm on the left parietal (Figure 2). However, intraoperatively, the dura was not involved with the tumor; hence, it did not require any duraplasty.

**Figure 3:**
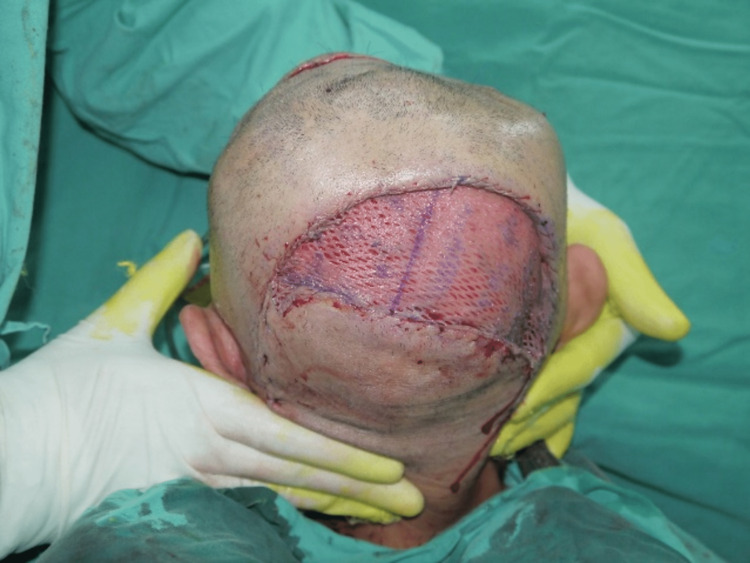
Visor flap covering both skull defects. Secondary donor defect grafted with a split skin graft.

Given that the defect was huge and multiple, the bi-pedicle flap raised based on bilaterally superficial temporal arteries, including posterior auricular arteries, was reliable enough to perfuse the flap. The flap was raised via the plane of the sub-galea. The galea was scored to increase the mobility of the flap. Eventually, the defects could be covered entirely, and the secondary donor defect was simultaneously grafted with a split skin graft (Figure 3). Drains were inserted to prevent hematomas.

**Figure 4:**
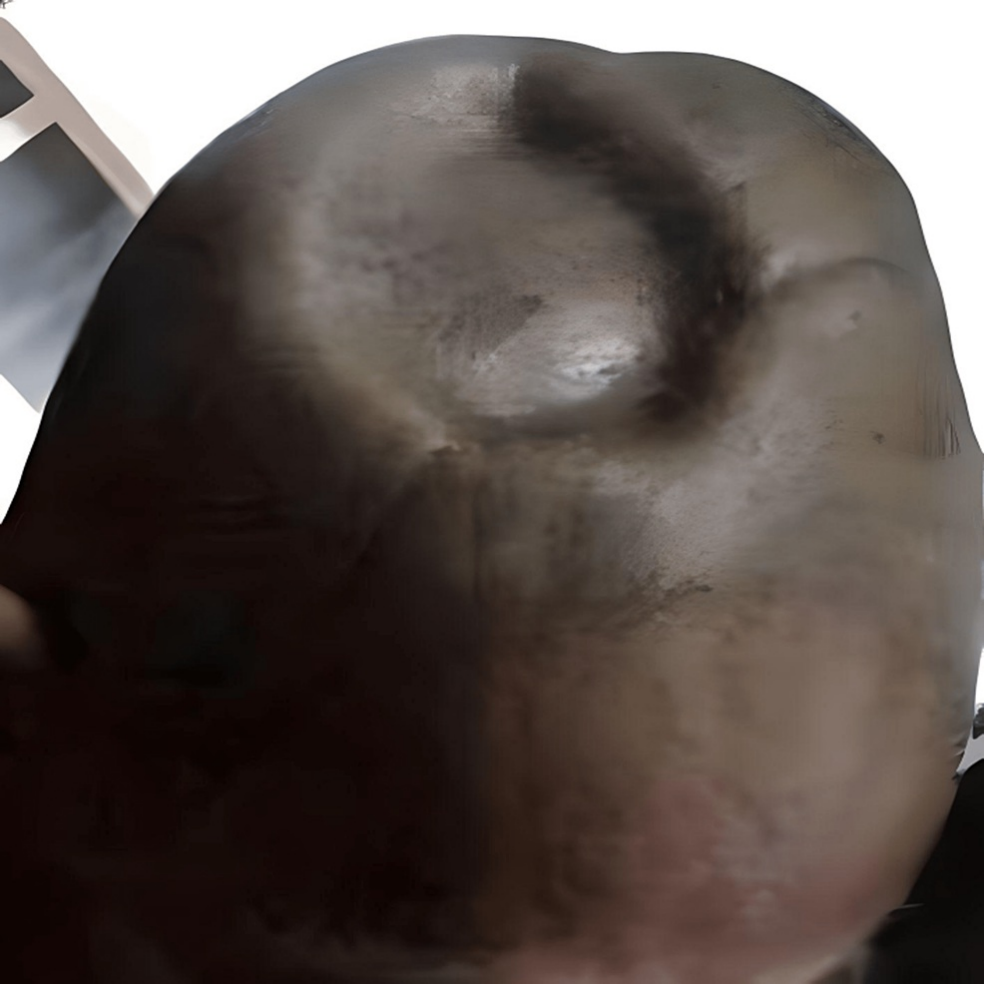
One month postoperatively, the flap covered the defect well with no wound dehiscence. The contour deformity was prominent due to a skull defect underneath.

The flap was well-perfused post-surgery, and the patient was discharged home. One month postoperatively, the flap covered the defect well with no wound dehiscence, even though the contour deformity was prominent due to two skull defects underneath (Figure 4). Unfortunately, due to disease progression, the patient succumbed to the disease three months after the surgery.

## Discussion

Modern reconstructive surgery is safe enough to be used even on elderly or ill patients. It permits the extensive excision and restoration of any extent of defects in scalp malignancy [2,3]. The closure of scalp defects is influenced by several elements, including the size and location of the defect, the quality of the remaining scalp tissue, and the hair-bearing significance [2]. With the limitations of options due to tissue elasticity, underlying skull convexity, and the hairline’s significance, some authors have provided algorithms for managing scalp defects [3]. After the resection achieves a clear oncologic margin, primary closure is possible for defects smaller than 4 cm [3]. Skin grafts can be an option if the pericranium or galea are still preserved after the tumor excision [3]. However, consideration needs to be taken when the coverage is at the hair-bearing area. Skin grafts often result in instability, contour deformity, or unpleasant scars with poor functional and aesthetic outcomes [2]. In such a case, a secondary procedure such as tissue expansion or serial excisions is needed to excise the alopecic areas [3].

Nevertheless, for more extensive defects, a free flap is considered the first choice for reconstructing large or composite defects involving the bone, soft tissue, and skin [4]. These options must be according to the reconstruction goals, whether to restore integrity, functions, or forms [4]. When considering the best reconstructive options, the goals of the reconstruction alone or in combination will depend on the patient’s state of health, comorbidities, and wishes [4].

In our case, the goal was mainly to restore tissue integrity, with less consideration given to restoring function and aesthetic appearance. After considering the nature of the disease and the patient’s health status, the option of a loco-regional flap was considered to minimize the complications of long surgery and anesthesia. Moreover, in metastatic scalp lesions, the risk of recurrence is significant; hence, secondary surgery is later preferable for free tissue transfer [4]. Various local flap designs are practically used in scalp reconstruction, and the flap choice greatly depends on the surgeon’s preference. Defects in the parietal scalp are amenable to adjacent tissue reconstruction. In addition, the soft tissue in the parietal area is more flexible than elsewhere due to the distinctive layer of temporoparietal fascia that overlies the deep temporal fascia instead of the periosteum [5], which allows the parietal scalp tissue to advance more if loco-regional flaps are used. In large parietal scalp defects of 25 cm^2^ or greater, bi-pedicled scalp flaps (known as visor flaps) have been used to close these defects. This flap is the best option for a single-stage reconstruction when the best cosmetic outcome is not a concern [6].

The visor flap offers a novel method for closing complex scalp defects. Jadhav et al. [7] described using bi-pedicled scalp flaps to restore calvarium injuries following high-tension electric burns. They offered well-vascularized tissue coverage of large, full-thickness wounds involving the scalp, calvarium, dura, and brain necrosis [7]. Hwang et al. [2] reported the largest defect size of 5 cm for the visor flap reconstruction, mostly to cover the skull defect after craniotomies. The bi-pedicled scalp flap is well perfused because it preserves the bi-directional flow via choke anastomosis, allowing large flaps to survive [8]. Therefore, it can be raised over a vast surface area without compromising tissue perfusion.

Furthermore, by undermining adjacent tissue, this flap can reconstruct scalp defects up to 25 cm × 20 cm in size [2]. The drawback of this visor flap is the needed skin grafting of the donor defect, especially in the coverage of large defects, which some modifications can overcome. The V-V modification reported by Hwang et al. [2] enabled the advancement of the scalp at the donor site over the convex skull, precluding the need for additional skin graft reconstruction for donor site coverage. The most common complication of scalp reconstruction is hematoma formation [1]. A proper history of blood-thinning drugs, including herbals, must be identified and stopped. Hemostasis needs to be done cautiously, as extensive cauterization of hair follicles can produce areas of permanent alopecia.

## Conclusions

Scalp reconstruction offers a great challenge to reconstructive surgeons. Factors such as limited tissue mobility, hairline respect, and the skull’s convexity compel a proper tissue reconstruction plan. Considering the best flap is subjective and according to the surgeon’s preference. The bi-pedicled visor scalp flaps are a simple, effective, and reliable reconstructive option for large and multiple skull defects, notably in those unsuitable for lengthy free flap surgeries. It also has the advantage of like-with-like tissue replacement and can provide a pleasant cosmetic appearance. Nevertheless, tissue coverage of these calvarial defects with vascularized tissue is crucial to prevent secondary life-threatening infections and improve individuals’ quality of life.

## References

[1] Angelos PC, Downs BW (2009). Options for the management of forehead and scalp defects. Facial Plast Surg Clin North Am.

[2] Hwang L, Ford NK, Spitz J, Ellis M (2017). The visor flap: a novel design for scalp wound closure. J Craniofac Surg.

[3] Iblher N, Ziegler MC, Penna V, Eisenhardt SU, Stark GB, Bannasch H (2010). An algorithm for oncologic scalp reconstruction. Plast Reconstr Surg.

[4] Chim H, Salgado CJ, Seselgyte R, Wei FC, Mardini S (2010). Principles of head and neck reconstruction: an algorithm to guide flap selection. Semin Plast Surg.

[5] Leedy JE, Janis JE, Rohrich RJ (2005). Reconstruction of acquired scalp defects: an algorithmic approach. Plast Reconstr Surg.

[6] De Haro F, Giraldo F (2001). Bipedicled fronto-occipital flap for reconstruction of postoncologic defects of the lateral scalp. Plast Reconstr Surg.

[7] Jadhav CN, Kumar Sharma R (2014). Bipedicled scalp flaps for reconstruction of high-tension electric burns of calvarium. J Craniofac Surg.

[8] Marty F, Montandon D, Gumener R, Zbrodowski A (1986). Subcutaneous tissue in the scalp: anatomical, physiological, and clinical study. Ann Plast Surg.

